# Canola Meal versus Soybean Meal as Protein Supplements in the Diets of Lactating Dairy Cows Affects the Greenhouse Gas Intensity of Milk

**DOI:** 10.3390/ani11061636

**Published:** 2021-05-31

**Authors:** Lucia Holtshausen, Chaouki Benchaar, Roland Kröbel, Karen A. Beauchemin

**Affiliations:** 1 Lethbridge Research and Development Centre, Agriculture and Agri-Food Canada, Lethbridge, AB T1J 4B1, Canada; lucia.holtshausen@canada.ca (L.H.); roland.kroebel@canada.ca (R.K.); 2 Sherbrooke Research and Development Centre, Agriculture and Agri-Food Canada, Sherbrooke, QC J1M 0C8, Canada; chaouki.benchaar@canada.ca

**Keywords:** agriculture, carbon footprint, dairy, enteric methane, greenhouse gas emissions, life cycle assessment, mitigation

## Abstract

**Simple Summary:**

Soybean meal (SBM) and canola meal (CM) are routinely used as protein supplements in lactating dairy cow diets and an enteric methane-mitigating effect was recently reported for CM compared with SBM. Farm-gate life cycle assessments of milk production in eastern and western Canada were conducted to determine whether using CM rather than SBM in lactating dairy cow diets decreases greenhouse gas emissions (CO_2_e) per kilogram of fat and protein corrected milk (GHG intensity), and whether the decrease in emission intensity of milk depends upon where the meals are produced. We concluded that protein source, location of producing the meals, and the methane-mitigating effect of CM influenced the GHG intensity of milk. CM was a GHG mitigation strategy, lowering GHG intensity of milk by up to 6.6% when it was produced in western Canada, with a low up-stream emission factor and a methane mitigating effect (i.e., low Y_m_ value). We conclude that the emissions associated with imported protein sources affect whether they decrease the GHG intensity of milk production when included in lactating cow diets.

**Abstract:**

Soybean meal (SBM) and canola meal (CM) are protein supplements used in lactating dairy cow diets and, recently, an enteric methane-mitigating effect (i.e., lower Y_m_ value) was reported for CM. Before recommending CM as a greenhouse gas (GHG) mitigation strategy, it is necessary to examine the net impact on total GHG emissions from milk production. The objective was to determine whether using CM rather than SBM in lactating dairy cow diets decreases GHG per kilogram of fat and protein corrected milk (FPCM), and whether the decrease depends upon where the meals are produced. Cradle to farm-gate life cycle assessments were conducted for a simulated dairy farm in eastern (Quebec) and western (Alberta) Canada. Scenarios examined the source of protein meal, location where meals were produced, and the methane-mitigating effect of CM. The Holos model was used to estimate GHG emissions from animals, manure, crop production, imported feeds, and energy use. GHG intensities (CO_2_e/kg FPCM) were 0.85–1.02 in the east and 1.07–1.11 in the west for the various scenarios, with enteric methane comprising 34 to 40% of total emissions. CM produced in western Canada with a low up-stream emission factor and low Y_m_ value reduced CO_2_e/kg FPCM by 3% (western farm) to 6.6% (eastern farm) compared with SBM. We conclude that using CM rather than SBM in the diet of lactating dairy cows can be a GHG mitigation strategy depending upon where it is produced and whether it decreases enteric methane emissions.

## 1. Introduction

Milk is recognized as being highly nutritious, with global yearly consumption estimated at 113 kg of raw milk per capita in 2018 [1]. However, there is increasing concern over the environmental impact of food production and, consequently, dairy supply chains worldwide have committed to lowering greenhouse (GHG) emissions to meet the commitments of the Paris Agreement. Many milk processing companies worldwide (e.g., Fonterra, New Zealand; Nestlé, Switzerland; Arlo Foods, Denmark; First Milk, United Kingdom; Emmi AG, Switzerland) have pledged to reduce GHG emissions at the farm level. In the United States, the Net Zero Initiative by a group of dairy industry stakeholders, including farmers, cooperatives, processing companies, and industry organizations, is helping dairy farms decrease GHG emissions by promoting optimized feed production, enhanced feed efficiency, decreased enteric methane (CH_4_) emissions, improved manure and nutrient management, and energy generation [2].

Gerber et al. [3] reported a global average GHG intensity of 2.4 kg carbon dioxide equivalent (CO_2_e)/kg of fat and protein corrected milk (FPCM), with CH_4_, nitrous oxide (N_2_O), and carbon dioxide (CO_2_) from animals, manure, feed production, and fuel use included in the life cycle assessment from “cradle to retail”. The GHG intensity of milk varied greatly among different regions of the world, ranging from 1 to 2 kg CO_2_e/kg FPCM for the industrialized regions of the world, to between 3 and 5 kg CO_2_e/kg FPCM for South Asian, West Asian, North African, and Central and South American countries, with a high of 7.5 kg CO_2_e/kg FPCM for sub-Saharan Africa [3]. More than 70% of the GHG emissions from milk production occurs prior to the farm-gate [4], with enteric CH_4_ comprising 35% to 55% [4,5,6] of farm emissions. Therefore, abatement of farm-based emissions significantly reduces the GHG emission intensity of milk.

Diet formulation shows promise in terms of mitigating enteric CH_4_ emissions [7,8]. However, a change in ingredient composition of diets to lower CH_4_ emissions may also alter the GHG emissions from feed produced on- and off-farm, as well as emissions from transportation of imported feed to the farm. As the GHG emissions associated with feed production and transportation comprise almost 30% of GHG intensity of milk production to the farm-gate in industrialized countries [4], it is important to understand the implications for GHG intensity of milk when recommending changes in diet composition to decrease enteric CH_4_ production.

Soybean meal (SBM), which contains 50 to 55% crude protein (CP) on a dry matter (DM) basis, is the most widely used protein source in livestock diets globally. Canada produces approximately 1.5 million tonnes of soybean meal per year, which is used as livestock feed [9]. Additionally, Canada is a major producer of canola seed, with 8.4 million ha planted in 2020 (55% in Saskatchewan (SK)), resulting in 5.8 million tonnes of meal for livestock feeding [10]. Canola meal (CM), which contains 40.9% ± 2.8% CP (DM basis) [11]), can be a cost-effective high quality protein source for dairy diets. Dairy diets are supplemented with protein to meet the nitrogen requirement of the rumen microbes and the amino acid requirements of the cow. In a recent study, Benchaar et al. [12] concluded that replacing solvent-extracted SBM with solvent-extracted CM in iso-nitrogenous diets of lactating dairy cows decreased enteric CH_4_ emissions expressed relative to gross energy (GE) intake (i.e., Y_m_ value) by 13%.

Changes in diet formulation may alter milk production of cows, and because GHG intensity is calculated as the ratio between emissions and product (i.e., meat and milk), an increase in animal performance could lower the GHG intensity of milk [13]. At low dietary inclusion rates (<10% of dietary DM) in iso-nitrogenous diets, no difference in dairy cow performance was observed between SBM and CM [14,15,16]. However, greater CM incorporation may increase milk production; Benchaar et al. [12] reported that milk production increased linearly as CM proportion in the diet increased up to 24% of DM.

While CM is routinely used as a protein source in dairy diets to promote milk production, prior to recommending its use to decrease enteric CH_4_ production, it is important to conduct a farm-scale life cycle assessment that considers all emission sources to determine the net effects on the GHG intensity of milk. Mathematical models that estimate whole farm GHG emissions can be useful tools for assessing changes in farm management practices. The whole-farm model and software tool Holos, developed by Agriculture and Agri-Food Canada, has been used previously to estimate GHG emissions from Canadian dairy [5,17] and beef [18] farms.

The objective of the present study was to determine whether feeding CM rather than SBM to lactating dairy cows should be recommended as a GHG mitigation strategy. The study focused on Canada because dietary mitigation strategies are dependent upon local conditions, although the concepts and methodology used are universally applicable for evaluating GHG mitigating strategies. We compared the use of SBM and CM as protein supplements in the diets fed to lactating cows on the GHG emission intensity of milk production for a representative dairy farm in eastern (Quebec, QC) and western (Alberta, AB) Canada. The net impact of the CH_4_-mitigating property of CM compared with SBM as reported by Benchaar et al. [12] was examined. We also considered whether the place of origin of the SBM and CM within Canada affects the GHG emission intensity of milk.

## 2. Materials and Methods

Cradle to farm-gate life cycle assessments were performed to evaluate the effects of using CM rather than SBM in the diet of lactating dairy cows on GHG emission intensity of milk produced in QC and AB, Canada. The primary data for the QC farm were from Benchaar et al. [12], while Oba et al. [19] provided the data source for the AB farm. Because CM and SBM purchased by farms in QC can be produced in the province (locally) or nationally (CM from SK; SBM from Ontario (ON)), the location effect of CM and SBM was considered in the QC dairy farm simulations. Five scenarios evaluated the source of protein meal used in the lactating cow diets as follows: (Q1) SBM from QC (local), (Q2) SBM from ON (national), (Q3) CM from QC (local) with enteric CH_4_ mitigation effect (CM_with_), (Q4) CM from SK (national) with enteric CH_4_ mitigation effect (CM_with_), and (Q5) CM from QC (local) without enteric CH_4_ mitigation effect (CM_without_). The origin of the protein meals used by AB dairy farms is less variable; therefore, the scenarios were as follows: (A1) SBM from ON, (A2) CM_with_ from AB, and (A3) CM_without_ from AB.

The study simulated a representative dairy farm in QC and in AB, each with herd size, diets, and management conditions typical of the region. As animal numbers and categories on a dairy farm fluctuate over time, the analysis was performed by considering the entire lifespan of an average dairy cow and its off-spring to account for the herd dynamics, similar to the approach used previously [5,17].

### 2.1. Quebec Dairy Farm (Eastern Canada)

The simulated dairy farm was located in Sherbrooke, QC, within Ecodistrict 483 in the Atlantic Maritime Ecozone. Average precipitation and evapotranspiration during the growing season (May to October) in this ecozone are 550 and 586 mm, respectively [20], and the soil classification is Podzolic with medium texture [21]. The milk production and CH_4_ emission data for the lactating cows were from the study of Benchaar et al. [12], which was conducted at the Sherbrooke Research and Development Centre of Agriculture and Agri-Food Canada in the same region as the simulated farm.

#### 2.1.1. Herd Dynamics, Housing, and Manure Storage

**Figure 1:**
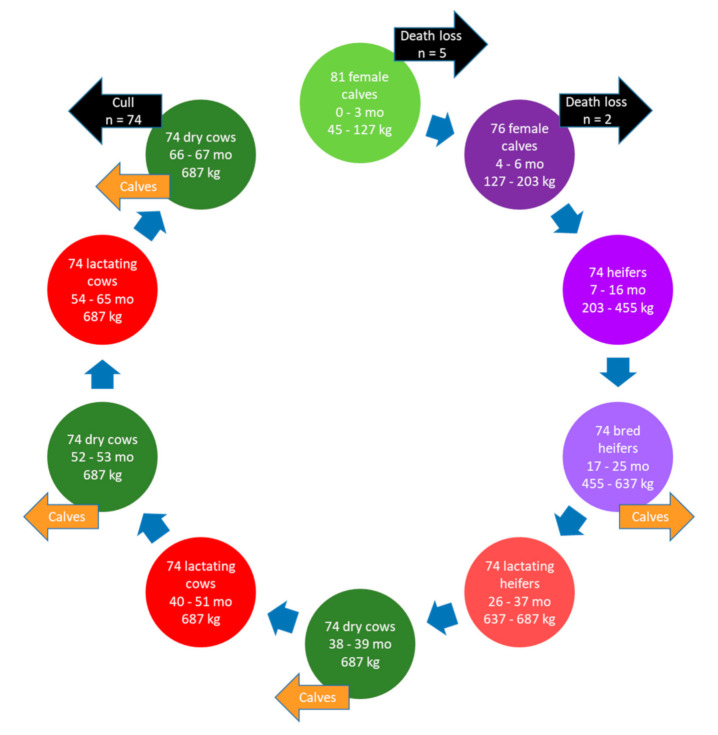
Life cycle of a dairy herd in Quebec illustrating the herd dynamics over time for dairy cows with a productive lifespan of three lactations.

The farm had a herd size of 74 lactating cows, which is the average for dairy farms in this region [22]. The analysis incorporated all dairy animals on the farm, including the pre-weaned and weaned calves, replacement heifers to the point of first calving, lactating dairy cows over multiple lactations, dry cows, and veal calves used to produce meat from calves not used as replacements (Figure 1). To achieve the desired herd size, the analysis started with the birth of 81 female calves to account for the average mortality rate of 6.4% for pre-weaned (0 to 3 months of age) and 2.4% for weaned (4 to 6 months) calves [23]. Inputs for average body weight at different life stages, age at first calving, calving interval, and dry period duration were based on statistics for the province of QC [22]. The lifespan of the dairy cows encompassed three lactations based on the average culling rate for Canadian dairy cows (32.7%) [24]. The productive lifespan was calculated as the reciprocal of the culling rate (i.e., productive lifespan = 1/culling rate) [25]. All lactating cows were culled and sent to slaughter after the third lactation, dry period, and calving at 67 months. While this scenario represents the average practice, in reality, the cows would be culled throughout the cycle, but the outcome would be the same. The cycle finished 7 months later when the last group of veal calves were sold.

Accounting for a stillbirth rate of 4.9% [23], 70 live calves were born at each of four calving events, for a total of 280 calves during the 67-month cycle. A sex ratio of 51:49 male/female was assumed for calves. After accounting for female calves considered as replacements for the continuation of the herd, and pre-weaning and post-weaning mortality, 188 male and female calves entered the veal system at 3 months of age and 184 veal calves were slaughtered at 7 months, weighing 307 kg.

Lactating cows were housed in individual tie-stalls [26], bedded with straw, and manure was regularly removed and stockpiled (solid storage). All calves, young heifers, and dry cows were group housed in deep-bedded pens with straw bedding. All manure was spread annually on the farm’s land base.

#### 2.1.2. Diet Composition and Milk Production

The diet composition (Table 1) for the lactating cows was from Benchaar et al. [12]. Two iso-nitrogenous diets (16.35% CP) were evaluated (DM basis): (1) a diet containing 17% SBM and 0% CM, and (2) a diet containing 24% CM and 0% SBM. Soybean hulls were added to the SBM diet to ensure similar fiber content of both diets, while proportions of other ingredients were the same for both diets. Diets for heifers aged 7 to 25 months were based on typical diets fed at the Research Centre. All other diets (calves aged 0 to 6 months and veal) were formulated to meet total digestible nutrient and CP requirements [27]. Dry matter intake, milk production, and milk composition for mature cows (Table 2) were based on measurements [12]. Milk production for first lactation cows was estimated at 93% of that of mature cows [17], whereas milk composition was considered similar to that of mature cows.

#### 2.1.3. Feeds Grown on the Farm and Purchased

The forages used in the rations were grass/legume silage, corn silage, and timothy hay, and it was assumed that these were grown on the farm to meet the herd’s needs with no excess. The area of each crop was based on animal feed intake; crop yield; and losses associated with harvest, storage, and feed wastage. Yield, fertilizer rates, and lime application rates for the grass/legume and corn silage were based on unpublished data collected for the study reported by Benchaar et al. [12], and rates representative of the region were used for the alfalfa hay (Table 3). All crops were grown with no irrigation and reduced tillage. The grass/legume silage and alfalfa hay were assumed to be from a four-year perennial stand. All other feed ingredients were purchased and imported onto the farm assuming no losses or wastage (Table 4).

### 2.2. Alberta Dairy Farm (Western Canada)

The farm was situated near Picture Butte, AB within Ecodistrict 793 in the Prairies Ecozone, with average precipitation and evapotranspiration during the growing season (May to October) of 277 and 653 mm, respectively [20]. The soil classification was a dark brown Chernozemic with a medium texture [21]. The input data for the mature lactating cows were based on a lactation study [19] conducted at the University of Alberta Dairy Research and Technology Centre (Edmonton, AB, Canada).

#### 2.2.1. Herd Dynamics, Housing, and Manure Storage

**Figure 2:**
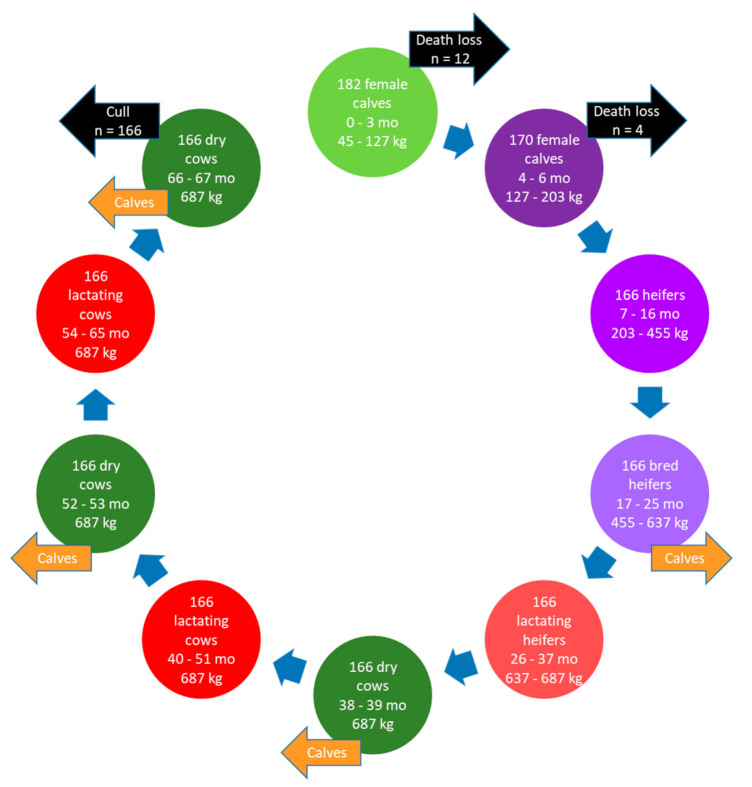
Life cycle of a dairy herd in Alberta illustrating the herd dynamics over time for dairy cows with a productive lifespan of three lactations.

The herd life cycle (Figure 2) started with the birth of 182 female calves to achieve a herd size of 166 lactating cows [22]. Inputs for average body weight at different life stages, age at first calving, calving interval, and dry period duration were based on industry statistics for AB [22]. The lactating cows were culled and sent to slaughter at 67 months and the cycle finished 3 months later when the last group of calves were weaned and sold to a feedlot (419 male and female calves sold total). This scenario represented the average for AB farms, although, in reality, the lifespan of individual animals would be variable. This approach simplifies the calculations, but does not affect the end results.

Lactating cows were housed in a free-stall housing system, as is typical for farms in AB [26]. The stalls were bedded with chopped straw and manure was regularly removed and stockpiled (solid storage). All calves, young heifers, and dry cows were group housed in deep-bedded pens with straw bedding. All manure was spread on the farm’s lands annually.

#### 2.2.2. Diet Composition and Milk Production

The lactating cow diets contained either 10% SBM or 13% CM (DM basis), with diet composition (Table 5) from Oba et al. [19]. Beet pulp and urea were added in different proportions to both diets to make the diets isonitrogenous, while both diets contained similar proportions of forages, grains, minerals, and vitamins. Diets for calves aged 0 to 6 months, heifers aged 7 to 25 months, and dry cows were formulated to meet total digestible nutrient and CP requirements [27]. Dry matter intake, milk production, and milk composition for mature cows (Table 2) were based on measurements [19]. Milk production for first lactation heifers was estimated at 93% of that of mature cows [17], whereas milk composition was considered as similar to that of mature cows.

#### 2.2.3. Feeds Grown on the Farm and Purchased

Barley grain and all forages, including grass hay, alfalfa hay, and barley silage, were grown on the farm, as is typical of farms in southern AB. The area of each crop was estimated in the same manner as for the QC farm. All crops were grown with irrigation and reduced tillage and yield and fertilizer rates were based on statistics for the region (Table 3). The grass and alfalfa hays were assumed to be from three-year perennial stands. All other feed ingredients were purchased and imported onto the farm, assuming no losses or wastage (Table 4).

### 2.3. GHG Emission Quantification

The GHG emissions for both farms were estimated using the research version of the Holos model 3.06 with the farm-gate as the system boundary [28]. The model is designed as an exploratory tool for producers, calculating emissions with Canada-specific emission factors and algorithms, accounting for all on-farm, as well as up-stream, emissions of CO_2_, CH_4_, and N_2_O. To simplify its utility, the model does not provide productivity estimates, and rather calculates emissions based on producer supplied values of crop yields and animal performance, from which biomass amounts (and N contained within) and DMI are calculated. All emissions were expressed as CO_2_-equivalents (CO_2_e) using the 100-year global warming potential of CO_2_ = 1, CH_4_ = 28, and N_2_O = 265 [29]. The emissions included were as follows: (1) CH_4_ from enteric fermentation and manure management; (2) direct and indirect nitrous oxide (N_2_O) from soils, cropping, and manure management; (3) CO_2_ from energy use; and (4) CO_2_e emissions associated with inputs (lime, fertilizer, herbicide, and feeds) imported onto the farm.

#### 2.3.1. Animal and Manure Emissions

Enteric CH_4_ emissions were estimated as a function of DMI and CH_4_ conversion factor (Y_m_, % of gross energy intake) for the diets (Table 1 and Table 5). The DMI for each animal category, with the exception of calves (0 to 3 months) and heifers (4–6 months), was estimated based on the net energy requirement for maintenance, activity, growth, pregnancy, and lactation, and the estimated net energy content of the diets [27]. The DMI for calves (0 to 3 months) and heifers (4–6 months) was based on recommended intake levels to meet growth requirements [27]. For the QC farm, the Y_m_ values for the lactating cows were from Benchaar et al. [12], with emissions measured in respiratory chambers. For scenario Q5, the same Y_m_ value was used for both lactation diets, thereby removing the CH_4_ mitigation effect of CM. As enteric CH_4_ production was not measured in the study by Oba et al. [19], the Y_m_ values for the AB lactating cows were based on IPCC [29] with consideration for geographical location, animal category, and dietary forage proportion. This approach resulted in a single Y_m_ value for both lactation diets. Therefore, to evaluate the potential CH_4_ mitigation effect of CM for the AB farm, an additional scenario was conducted (AB2) in which the Y_m_ value for CM diet was decreased by 13%, reflecting the mitigation effect of CM reported by Benchaar et al. [12]. All other Y_m_ values were based on IPCC [29] according to animal category and dietary forage proportion.

The quality of manure (volatile solids) produced was estimated from DMI and digestibility of the diets. Emissions from manure management were then calculated by multiplying volatile solids production by the CH_4_ producing capacity of the manure (Bo = 0.24 for lactating dairy animals, Bo = 0.19 for all other animal categories) and CH_4_ conversion factor (MCF) of the manure handling system. The MCF is 0.002 for solid storage and 0.17 for deep bedding [29].

To calculate N_2_O emissions from manure, the quantity of N excreted in manure was estimated based on DMI, CP concentration of the diet, and N retention of cattle [29]. Direct N_2_O emissions were estimated by multiplying the quantity of manure N excreted by an emission factor specific to the manure handling system (solid storage = 0.005; deep bedding = 0.01) [29]. Indirect N_2_O emissions from leaching, runoff, and volatilization were estimated based on fractional manure losses and estimates of the N content of manure (Table 4).

The emissions of CO_2_ associated with energy use were calculated based on the estimated electricity usage of 968 kWh per dairy cow per year (derived from [31]). The volume and concentration of manure produced and an emission factor of 0.347 kg CO_2_e/kg manure N were used to estimate emissions of CO_2_ associated with diesel fuel use for spreading manure [28].

#### 2.3.2. Crop Production and Imported Feed Emissions

Emissions of CO_2_ from energy used for crop production included those associated with N and phosphorus fertilizer production; herbicide production; and production, transport, and degradation of lime (Table 4). Direct N_2_O emissions from soils and cropping were based on N inputs from synthetic N fertilizer, above- and below-ground crop residue decomposition, and land applied manure. Crop residue N input was calculated [28] based on crop yields and coefficients from Janzen et al. [37] (Table 3). Below-ground residue input for perennial crops was calculated assuming 39% of root biomass turnover annually and complete return-to-soil in the final year [38]. The modified impact on these N inputs due to soil texture, climate, and tillage [34] was incorporated within the Holos simulation [31]. Indirect N_2_O emissions due to leaching, runoff, and volatilization were based on loss fractions and indirect emission factors [29]. Changes in soil carbon stocks were not considered in the analysis, as it was assumed that any change would be similar for the CM and SBM scenarios.

To estimate the CO_2_e from importing feeds onto the farm, a published emission factor for each feed was used and the energy used to transport the feed by truck and rail from the nearest site of manufacture to the farm was included. For the QC farm, the locally produced SBM (and hulls) and CM were assumed to be transported by truck from a processing plant in Bécancour, QC to the farm (150 km). For the nationally sourced meals, SBM (and hulls) was transported by truck from Windsor, ON (1000 km), while CM was transported by rail from Saskatoon, SK to Montreal, QC (3000 km) and then by truck from Montreal, QC to the farm (160 km). For the AB farm, the CM was assumed to be from locally grown canola processed in Lethbridge, AB and transported to the farm by truck (30 km), while SBM and hulls were transported by rail from Windsor, ON to Edmonton, AB (2600 km) and then by truck from Edmonton, AB to the farm (500 km).

The rail emission factor was calculated using the average rail fuel consumption (L/km) [39] multiplied by the rail diesel combustion emission factor from Canada’s National GHG Inventory Report [40]. The truck emission factor was calculated based on an average truck fuel consumption of 2.48 km/L [41] multiplied by the heavy-duty diesel vehicle combustion emission factor from Canada’s National GHG Inventory Report [40].

#### 2.3.3. Functional Unit and Co-Product Allocation

The GHG emissions were expressed relative to multiple functional units for the comparison of scenarios: kg of FPCM (standardized to 4% fat and 3.3% protein), kg of meat on a live weight basis, kg of meat on a carcass weight basis, kg of protein, and MJ of energy.

The calculation of FPCM was as follows [42]:

FPCM (kg/yr) = milk (kg/yr) × [0.1226 × fat (%) + 0.0776 × true protein (%) + 0.2534](1)

Carcass weight was calculated based on a dressing percentage of 60% of live weight [5]. The assumed protein content of meat was 17.32% of carcass weight and its energy value was 12.18 MJ/kg carcass [43]. The energy value of milk [27] was calculated as follows:

Milk energy (MJ/kg) = 4.184 × [0.0929 × fat (%) + 0.0563 × true protein (%) + 0.192](2)

Physical allocation [42] was used to calculate the allocation factor for milk (AFmilk) and meat (AFmeat):

AFmilk = 1 − 6.04 × Mmeat/Mmilk and AFmeat = 1 − AFmilk(3)

where Mmeat is the total live weight of all animals sold and Mmilk is the total weight of FPCM sold.

### 2.4. Sensitivity Analysis 

To determine the relative impact of several important sources of emission, a sensitivity analysis was conducted for the GHG intensity of FPCM (with allocation), as described by Rotz et al. [44]. The relative impact of the following inputs was compared: (1) decreased Y_m_ value of the lactation diet, (2) decreased emission factors for imported SBM and CM, (3) increased FPCM production (assumptions: no change in DMI or manure; representing improved genetic selection of animals), and (4) decreased DMI of the lactating cows through increased total digestible nutrient content of feed (assumptions: no change in FPCM, less manure, less home-grown and imported feed, less land required; representing improved ration digestibility). The sensitivity index was calculated as the percentage change in GHG intensity of FPCM divided by a 10% change in the input variable [44]. The sensitivity index ranges from 0 to 1.0; an index near 0 indicates a minimal effect and an index near 1.0 means a large effect on the GHG intensity of milk. The sensitivity analysis does not consider interaction effects among input variables.

## 3. Results

### 3.1. Quebec Farm

Protein source in the lactation diets (SBM vs. CM), location at which the meals were procured (locally vs. nationally), and the CH_4_-mitigating effect of CM (CM_with_ vs. CM_without_) influenced the GHG intensity of FPCM (Table 6). When both meals were produced in QC, kg CO_2_e/kg FPCM was 7% less when SBM compared with the case when CM_with_ was used in the lactation diet (Q1 vs. Q3), and 11% less for SBM without the mitigation effect of CM (Q1 vs. Q5). Thus, the enteric CH_4_-mitigating effect of CM did not offset the greater emission factor of CM compared with SBM (1.23 vs. 0.56 kg CO_2_e/kg DM) for meals produced locally. Procuring SBM from ON versus QC had only a very small (+1%, Q1 vs. Q2) effect on kg CO_2_e/kg FPCM. However, procuring CM nationally decreased the emission intensity of milk by 6.6% compared with using locally sourced SBM (Q4 vs. Q1). This decrease was due to the low emission factor of CM procured from SK (0.29 CO_2_e/kg DM), even though emissions from feed transportation to the farm in QC increased.

The CO_2_e emissions from imported feeds were 60% greater (Q3 vs. Q4, 351 vs. 219 g CO_2_e/kg FPCM) when CM was from QC compared with SK (Table 7), owing to their respective emission factors (1.23 vs. 0.29 kg CO_2_e/kg DM). Although emissions from transportation of feed increased when importing CM from SK (+6 g CO_2_e/kg FPCM), the net impact on the GHG intensity of milk still favored the use of CM from SK. Furthermore, the emission factor for CM, which varied considerably with location, had a greater impact on kg CO_2_e/kg FPCM than the enteric CH_4_-mitigation effect of CM (Q3 vs. Q5, −39 g CO_2_e/kg FPCM). The combined effects of low enteric CH_4_ and low imported feed emissions accounted for Q4 (i.e., CM_with_ from SK) having the lowest emission intensity of FPCM for the QC dairy farm.

**Figure 3:**
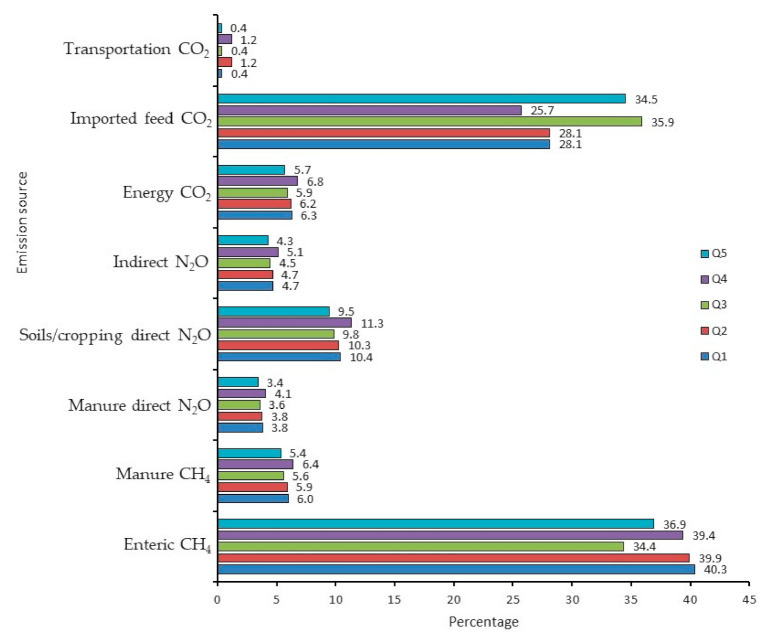
Greenhouse gas proportions of kg CO_2_/kg FPCM for dairy production systems using soybean meal (SBM) or canola meal (CM) in the diets for lactating cows for simulated dairy farms in Quebec. The scenarios were as follows: (Q1) SBM from QC, (Q2) SBM from ON, (Q3) CM from QC with enteric CH_4_ mitigation effect, (Q4) CM from SK with enteric CH_4_ mitigation effect, and (Q5) CM from QC without enteric CH_4_ mitigation effect.

Emissions from enteric CH_4_ and imported feeds had a substantial impact on CO_2_e per kg FPCM. Enteric CH_4_ was the largest source of emissions associated with milk production, comprising approximately 40% of the total CO_2_e per kg FPCM for all scenarios except Q3 (CM_with_ from QC), where enteric CH_4_ was 34% of the total emissions owing to the proportionally greater emissions from purchased feeds (Figure 3). Emissions from imported feeds varied from 25.7% (Q4) to 35.9% (Q3), increasing to 26.9% and 36.2%, respectively, when transportation of feed was included. Thus, transportation emissions associated with the protein sources were relatively small. Indirect and direct N_2_O emissions from on-farm crop production comprised 13.8% (Q5) to 16.4% (Q4) of the total CO_2_e per kg FPCM. Emissions from stored manure were relatively small: 3.4% (Q5) to 4.1% (Q4) for N_2_O, and 5.4% (Q5) to 6.4% (Q4) for CH_4_. Energy-associated emissions (not including transportation of feed) were also relatively small, at 5.7% (Q5) to 6.8% (Q4) of the total.

Physical allocation averaged 80.25% to milk and 19.75% to meat, with little variation among the scenarios (Table 6). Because allocation of emissions to meat and milk was similar for all scenarios, GHG intensities expressed relative to meat (live weight and carcass weight) followed the same trend as for FPCM. Similarly, using protein and energy as functional units resulted in the same ranking of the scenarios.

### 3.2. Alberta Farm

**Figure 4:**
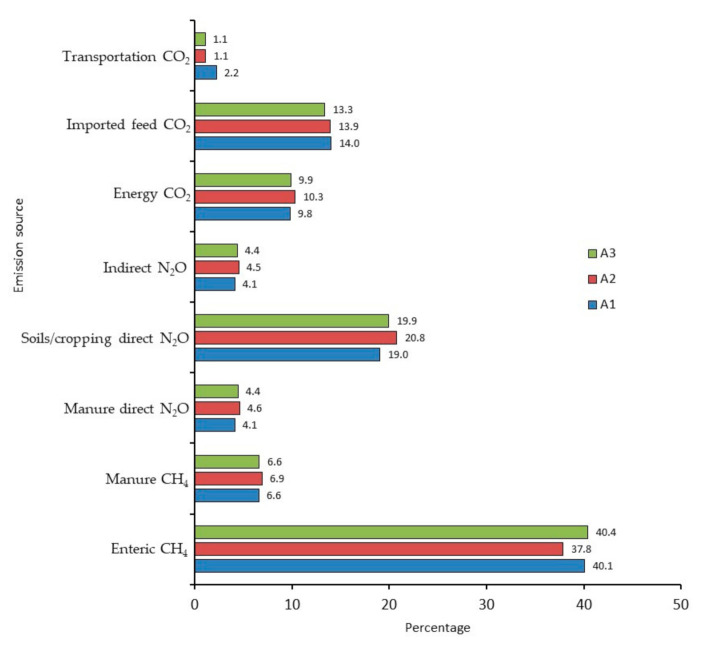
Greenhouse gas proportions of kg CO_2_/kg FPCM for a dairy farm in Alberta using soybean meal (SBM) or canola meal (CM) in the diets of lactating cows. The scenarios were as follows: (A1) SBM from ON, (A2) CM from SK with enteric CH_4_ mitigation effect, and (A3) CM from SK without enteric CH_4_ mitigation effect.

Protein source in the diets of lactating dairy cows had only marginal effects on the total CO_2_e per kg FPCM (Table 8). The kg CO_2_e/kg FPCM was similar for SBM and CM_without_ (A1 vs. A3, 1.10 vs. 1.11, respectively). Assuming a 13% lower Y_m_ value for CM_with_ than SBM, the GHG intensity of milk was 3% less (reduced to 1.07 kg CO_2_e/kg FPCM for A2) than that of SBM (A1). The decrease in the intensity of milk production was due solely to decreased enteric CH_4_ emissions, as other sources of emissions remained relatively stable (Table 9). Enteric CH_4_ was the greatest contributor to kg CO_2_e/kg FPCM, comprising 37.8% (A7) to 40.4% (A8) of total emissions (Figure 4). Emissions from imported feeds ranged narrowly from 13.3% (A3) to 14.0% (A1), increasing to 14.4% and 16.2%, respectively, when transportation of protein sources was included. Indirect and direct N_2_O emissions from on-farm crop production comprised 23.1% (A1) to 25.3% (A2) of the total CO_2_e/kg FPCM. Emissions from stored manure were relatively small: 4.1% (A1) to 4.6% (A2) for N_2_O, and 6.6% (A3) to 6.9% (A2) for CH_4_. Energy-associated emissions (not including transportation of feed) were also relatively small, at 9.8% (A1) to 10.3% (A2) of the total. The protein source in the lactation diets had minimal effects on the GHG emission intensity of the other functional units (Table 8).

### 3.3. Sensitivity Analysis 

**Figure 5:**
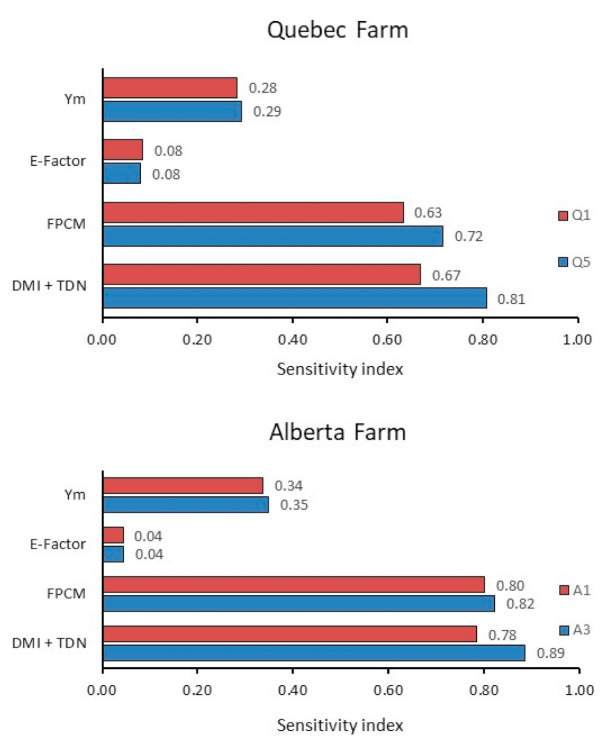
Sensitivity index showing the relative effect of input variables on the GHG intensity of FPCM for a Quebec (Q1, SBM from QC (local); Q5, CM from QC (local) without enteric CH_4_ mitigation effect) and Alberta (A1, SBM from ON; and A3, CM from SK without enteric CH_4_ mitigation effect) scenarios. An index near 0 indicates a minimal effect and an index near 1.0 means a large effect. The input variables were as follows: (1) decreased Y_m_ value of the lactation diet, (2) decreased emission factors for imported SBM and CM, (3) increased FPCM production, and (4) decreased DMI of the lactating cows through increased total digestible nutrient content of feed.

The sensitivity analysis provides insights into the relative influence of a change of similar magnitude in various factors on the GHG intensity of FPCM, when considered individually (Figure 5). For both farms, a 10% improvement in feed conversion efficiency, either through increased FPCM or decreased DMI due to improved digestibility, had a large impact on the GHG intensity of milk (index, 0.63 to 0.89). A 10% decrease in Y_m_ of the lactation diets had a moderate impact (0.28 to 0.35), while a 10% change in emission factor of the protein sources had a relatively minor impact (<0.1).

## 4. Discussion

Benchaar et al. [12] reported that feeding an isonitrogenous diet supplemented with CM (23.7% of DM) rather than SBM (17.0% of DM) to lactating dairy cows decreased the Y_m_ by 13%. The CH_4_-mitigating effect of CM was accompanied by a 7% increase in DMI and a 5% increase in FPCM. While Oba et al. [19] did not measure enteric CH_4_ production, they reported no difference in milk production and DMI for lactating cows fed diets containing CM (13.0% of DM) or SBM (10.2% of DM). Using these data to model the GHG intensity of meat and milk produced by typical dairy farms in QC and AB showed that whether CM should be recommended as a GHG mitigation strategy depended upon two main factors: firstly, the emission factor of the CM relative to that of SBM, which varied with where the crops were grown and the meals produced; and, secondly, the CH_4_-mitigating property of CM.

Herd size, dietary ingredients, housing, farm management, and environmental growing conditions of the farms simulated in the present study to evaluate CM as a GHG mitigation strategy differed substantially, but were typical of farms in these respective areas of the country. The AB farm grew barley grain in addition to the forages required for the herd and, therefore, purchased and imported less feed onto the farm. Consequently, the proportion of total GHG emissions from purchased feeds (plus transportation) was greater for the QC (range: 26.9% to 36.2%) versus AB (range: 14.4% to 16.2%) farm and, inversely, the proportion of total GHG emissions from on-farm feed production was greater in AB. However, for both farms, enteric CH_4_ was the greatest contributor to kg CO_2_e/kg FPCM, ranging from 34% to 40% for the various scenarios. Previous studies also report enteric CH_4_ as the greatest contributor to the total GHG emissions from milk production in intensive dairying (range of 31.7% to 57.3% for individual farms in ON) [45]).

After allocation to meat, the GHG intensities of milk to the farm-gate ranged from 0.85 to 1.02 kg CO_2_e/kg FPCM for the QC farm scenarios and from 1.07 to 1.11 kg CO_2_e/kg FPCM for the AB farm scenarios. The slightly greater emission intensity of the AB farm was partially attributed to the use of default Y_m_ values [29] to estimate CH_4_ emissions in the absence of measured values. The measured Y_m_ values from Benchaar et al. [12] used for the QC herd were less than the default values used for the AB herd. In addition, the feed conversion efficiency (kg milk/kg DMI) was less for the AB herd compared with the QC herd. Improving feed conversion efficiency corresponds to a general trend for lower emissions per unit of milk produced [4].

The GHG intensity values reported in the present study are consistent with many other North American studies, despite differences in assumptions and methodologies. Gerber et al. [46] reported an average farm-gate emission intensity of 1.0 kg CO_2_e/kg FPCM for North America. Thoma et al. [4] and Capper et al. [47] reported national averages of 1.23 (2008) and 1.35 (2007) kg CO_2_e/kg FPCM, respectively (farm-gate), in the United States, while for the Canadian dairy industry (farm-gate), Vergé et al. [29] reported an average GHG intensity of 1.0 kg CO_2_e/kg milk, with slightly greater intensity in AB compared with QC (1.05 vs. 0.97 kg CO_2_e/kg milk). However, the opposite regional trend was reported by Thivierge et al. [48], with intensities of 0.95 to 0.99 kg CO_2_e/kg FPCM for AB and 1.12 to 1.22 kg CO_2_e/kg FPCM for QC. Jayasundara and Wagner-Riddle [49] reported an intensity of 1.03 kg CO_2_e/FPCM, ranging from 0.89 to 1.36 kg CO_2_e/kg FPCM, for ON (farm-gate) in 2011. A more recent survey of 142 farms in ON reported a range of 0.44 to 1.73 kg CO_2_e/kg FPCM, with a mean of 1.015 kg CO_2_e/kg FPCM [45].

It is often assumed that a decrease in the Y_m_ value of a diet, as reported by Benchaar et al. [12] for a lactation diet containing CM, would lower the emission intensity of milk production. However, a decreased Y_m_ value due to a change in ingredient composition of the diet may not lower total GHG emissions of milk if emissions elsewhere in the system increase. This concept is illustrated by the QC farm scenarios, whereby the enteric CH_4_-mitigating effect of CM did not offset the greater emission factor of CM compared with SBM when both meals were produced locally. Furthermore, using SBM from ON rather than QC did not alter the conclusion that, despite a CH_4_-mitigating effect of CM, SBM is a better choice than CM produced in QC in terms of minimizing the GHG emissions associated with milk and meat production. However, the comparison between using these protein sources in the lactation diets differed when CM from SK was used; its low up-stream emission factor caused a 6.6% decrease in GHG intensity of milk compared with using SBM produced in QC. This result highlights the importance of considering the source of CM and, consequently, its up-stream emission factor, prior to recommending it as a CH_4_ mitigation practice.

Emissions from imported protein meals include the CO_2_ from fossil fuel used in producing the crop (fertilizer, field operations, machinery use, and so forth), N_2_O emissions from the use of organic and inorganic fertilizers, carbon change in soils, and the CO_2_ from the fossil fuels used to process the seed and transport it to the farm. Emissions from transporting CM from SK to the farm in QC were small (1.2%, Figure 3) compared with the emissions associated with growing and processing the crop.

Globally, most SBM used in livestock feeding is imported from Argentina, Brazil, and the United States, with Canada being self-reliant in SBM. Using a consequential life cycle assessment, Dalgaard et al. [50] calculated that SBM produced in Argentina and delivered to the Netherlands had a global warming potential of 0.721 kg CO_2_e/kg if used to avoid palm oil and 0.344 kg CO_2_e/kg to avoid rapeseed oil, respectively. The emission factors for SBM and CM in the present study (Table 5) from Desjardins et al. [35] were derived using the traditional attributional approach, and thus not directly comparable to those reported by Dalgaard et al. [50]. Rotz et al. [51] assumed an emission factor of 0.37 kg CO_2_e/kg DM for SBM produced in the United States. In Canada, large differences in the emission factors of protein sources occur at the provincial scale, and these differences were shown in the present study to affect the GHG intensity of FPCM. Desjardins et al. [35] reported that the emission intensities in 2011 for canola seed on an area basis were 2700 kg CO_2_e/ha for QC and 530 kg CO_2_e/ha for SK. The lower emission intensity in SK is due to the high adoption rate of practices such as no-till and reduced summer fallowing that favor the sequestration of carbon in soils. Additionally, the drier climate in SK leads to lower N_2_O emissions, and large field sizes favor more efficient use of farm machinery and reduced fossil fuel consumption.

For the AB farm, the assessment of using CM rather than SBM as a protein source in lactation diets did not examine the location effect (emission factor) of producing the meals, because meals from multiple sources are typically not available in that region. The similar emission intensity of FPCM for SBM (A1) and CM_without_ (A3) scenarios indicates that, unless CM has a CH_4_-mitigating effect, there is no basis for recommending CM as a GHG mitigation strategy for dairy farms in AB, although its nutritional properties make it a valuable protein source for dairy cows [12,15]. When the CH_4_-mitigating property of CM was applied (AB2), the GHG intensity of milk decreased by 3% compared with that of SBM (A1) and by 4% compared with that of CM_without_ (A3). The decrease in the intensity of milk production when feeding CM_with_ was due solely to decreased enteric CH_4_ emissions, as other sources of emissions remained stable and milk production and DMI were unchanged.

The decrease in Y_m_ value when feeding CM to dairy cows reported by Benchaar et al. [12] needs further substantiation, as relatively few studies have examined the CH_4_ mitigation potential of protein sources. Gidlund et al. [52] reported a 6.6% numerical decrease in CH_4_ emissions (g/kg DMI) when feeding high proportions of CM (24% of DM) versus SBM (15% of DM) to dairy cows fed grass silage-based diets. The mitigation effect was attributed to a shift in rumen fermentation towards an increased molar proportion of propionate, an alternative hydrogen sink to CH_4_ in the rumen. However, Reynolds et al. [15] reported no effect on CH_4_ emissions when partially replacing SBM (9% of dietary DM) with CM (10% of diet DM) in the diet of lactating dairy cows. Thus, further research is needed before recommending feeding CM as a CH_4_-mitigating practice for milk production.

Flysjö et al. [53] indicated that the most important parameters affecting the GHG emissions of milk production were DMI and Y_m_, which affect the quantity of enteric CH_4_ produced, and the amount of N applied and the emission factor for direct N_2_O emissions from soils, which determine the N_2_O from crop production and contribute substantially to the emission factor of imported feeds. Our study shows that the emission factors for SBM and CM, as well as the Y_m_ value of the lactation diets, have significant effects on the GHG intensity of milk production, and the sensitivity analysis puts the relative impact of these factors into perspective. Although the sensitivity analysis examined only one factor at a time and did not consider interactions, it is considered a valuable tool in life cycle assessment [44].

According to the sensitivity analysis, a 10% decrease in Y_m_ of the lactation diet had a much greater impact on the GHG intensity of milk than a 10% decrease in the emission factor of the imported protein meals. However, for dairy farms, the emission factors of the imported protein meals vary substantially depending upon where the meals are produced, whereas the diet Y_m_ values vary only slightly. For example, the emission factor for CM in the present study varied by 424% (0.29 to 1.23 CO_2_e/kg DM) depending upon where it was produced, whereas the Y_m_ value of the lactation diets only varied by 13%.

Although the present study focused on the effects of protein source (emission factor of the meals and Y_m_ of the diet), the sensitivity analysis also included the impact of increasing milk production and improving feed conversion efficiency. It is well documented that improving animal performance decreases the GHG intensity of meat and milk production because fewer animals and less feed are needed to produce a certain amount of product [7]. Our analysis showed that, in comparison with the effects of Y_m_ value and protein source emission factor, changes in feed conversion efficiency due to increased milk production or decreased DMI (due to increased digestibility) clearly have larger potential to reduce the GHG intensity of milk, as indicated by sensitivity indices >0.63. Increased milk production can be achieved through genetic selection, nutrition, and management [54]. However, the impact of increased milk production in the present study is a “best-case” scenario, because it assumed no increase in DMI. In reality, increased milk production per cow is usually accompanied by more feed consumed per cow, but with a greater portion of the feed partitioned toward milk instead of maintenance and body growth. A decrease in DMI due to increased digestibility decreased the GHG intensity of milk because less feed was required and, therefore, emissions from the animal, manure, crops, and imported feeds were also decreased.

This LCA highlights the relative impacts of dietary protein supplements on the GHG intensity of milk by accounting for differences in up-stream emissions of producing SBM and CM and a potential enteric CH_4_-mitigating effect of CM. While the overall impact of the dietary protein source on the GHG intensity of milk was small (a decrease of 6.6% in the east and 3% in the west), it was not inconsequential. In comparison, the GHG intensity of milk produced in ON decreased by 1% annually over a 20-year period (2011 vs. 1991) owing to numerous improvements in production efficiency. On the other hand, it should be noted that there is considerable uncertainty associated with the inputs, farm characteristics, emission factors, and assumptions used to model the emissions in the present study, thus caution must be used when interpreting the results [44].

## 5. Conclusions

With increasing societal pressure on the dairy industry to lower its GHG emissions, dairy farmers need to understand the net impact of their reliance on purchased feeds on the total GHG emissions associated with milk production. Soybean meal and CM are both coproducts and important cost-effective protein supplements for dairy cows, but they also affect the GHG emissions of milk production. We conclude that using CM rather than SBM in the diet of lactating dairy cows can be a GHG mitigation strategy, lowering CO_2_e/kg FPCM depending upon where the CM is produced (which affects its global warming potential) and whether it has a CH_4_-mitigating property (the recently published CH_4_-mitigating effect of CM needs further substantiation).

For dairy farms in eastern Canada, using CM from western Canada decreased the GHG intensity of milk production by 6.6% compared with SBM by lowering the emissions from imported feeds, even though transportation emissions increased. In western Canada, using CM rather than SBM lowered the GHG intensity of milk by 3% if the CM had a CH_4_-mitigating effect. In both eastern and western Canada, the potential CH_4_-mitigating effect of CM compared with SBM decreased the GHG intensity of milk production, but further research is needed to substantiate this mitigation effect. While this study focused on milk production in Canada, the concepts and methodology used are transferrable and thus could be used elsewhere to evaluate GHG mitigating strategies.

## Figures and Tables

**Table 1:** Diet ingredients for each animal category for the simulated Quebec dairy farm.

Animal Category	Calves	Heifers	Veal	Heifers	Bred Heifers	Lactating Cows ^1^		Dry Cows
Age Group/Scenario	0–3 Months	4–6 Months	4–7 Months	7–16 Months	17–25 Months	SBM Diet	CM Diet	
Feed Ingredients, % of diet DM								
Grass/legume silage ^2^	-	-	20.0	55.0	50.0	25.0	25.0	-
Corn silage	-	-	-	30.0	-	21.9	21.9	10.0
Corn grain, cracked	10.5	45.0	70.0	8.00	3.00	14.7	14.7	-
SBM, solvent extracted	7.50	15.0	4.5	3.00	3.00	17.0	-	9.00
Soybean hulls	-	-	-	-	-	6.69	-	-
CM, solvent extracted	10.5	9.00	4.5	3.00	3.00	-	23.7	5.00
Timothy hay, chopped	-	30.0	-	-	40.0	5.21	5.20	70.0
Beet pulp, dehydrated	-	-	-	-	-	4.00	4.00	5.00
Rumen inert fat	-	-	-	-	-	1.99	1.99	-
Mineral/vitamin supplement	1.50	1.00	1.00	1.00	1.00	1.43	1.43	1.00
Calcium carbonate	-	-	-	-	-	1.41	1.41	-
Sodium bicarbonate	-	-	-	-	-	0.70	0.70	-
Milk replacer	70.0	-	-	-	-	-	-	-
Diet Characteristics								
DMI, kg/day ^3^	2.33	4.70	3.55	6.84	12.52	26.82 (26.02)	28.94 (28.05)	9.32
NE_L_, Mcal/kg DM ^4^	0.55	1.67	1.80	1.56	1.32	1.58	1.53	1.28
TDN, % of DM ^5^	85.2	73.6	78.0	68.0	59.0	69.0	67.0	57.0
Crude protein, % of DM ^6^	22.8	16.6	14.3	16.1	14.1	16.4	16.3	11.0
Y_m_, % gross energy intake ^7^	0.00	6.30	6.30	6.30	6.30	5.65	4.90	6.30

CM = canola meal; DM = dry matter; SBM = soybean meal. ^1^ Iso-nitrogenous lactating cow diets with SBM and SBM hulls or CM. ^2^ Mixture of timothy (70%) and red clover (30%). ^3^ Dry matter intake (DMI) for calves (0 to 3 months) and heifers (4 to 6 months) calculated based on intake requirements [27]; others estimated using Holos algorithms [28]; DMI for first lactation heifers in parenthesis. ^4^ Net energy of lactation for lactating cows from Benchaar et al. [12]; others calculated based on feed ingredient composition tables [27]. ^5^ Total digestible nutrient (TDN) for calves (0 to 3 months) and heifers (4 to 6 months) calculated based on feed ingredient composition tables [27]; others estimated using Holos algorithms [28]. ^6^ Crude protein for lactating cows from Benchaar et al. [12]; others calculated based on feed ingredient composition tables [27].^7^ Y_m_ for lactating cows from Benchaar et al. [12]; others based on IPCC [29].

**Table 2:** Average daily milk production and composition for the simulated dairy farms.

Milk Variables	Quebec Farm		Alberta Farm	
	SBM Diet	CM Diet	SBM Diet	CM Diet
Milk production—1st lactation, kg/day	37.6	40.4	33.7	32.9
Milk production—2nd and 3rd lactation, kg/day	40.4	43.4	36.2	35.4
Milk fat (all lactations), %	4.00	3.84	3.48	3.40
Milk protein (all lactations), %	3.42	3.38	3.21	3.25

SBM = soybean meal. CM = canola meal.

**Table 3:** Characteristics for farm-grown crops for the simulated dairy farms.

Item	Quebec Farm			Alberta Farm			
	Corn Silage	Grass/Legume Silage ^1^	Timothy Hay	Barley Grain	Barley Silage	Mixed Grass Hay	Alfalfa Hay
Agronomic Characteristics							
Yield, kg DM/ha	8200	3300	653	3382	5343	8130	8600
Irrigated	no	no	no	yes	yes	yes	yes
N fertilizer, kg N/ha ^2^	168	34	0	42	80	73	5.0
P fertilizer, kg P_2_O_5_/ha ^2^	85	21.5	0	25	25	25	25
Herbicide use ^2^	yes	no	no	yes	yes	no	no
Harvest/storage loss, % ^3^	12	12	12	3	12	12	12
Feed wastage, % ^3^	5	5	20	0	5.0	20	20
Moisture at harvest, % ^4^	34.6	32.5	13.0	9.0	64.5	55.0	69.0
Lime, kg CaCO_3_/ha ^5^	500	0	0	0	0	0	0
Relative DM Allocation ^4^							
Yield ratio	0.72	0.40	0.40	0.38	0.72	0.18	0.40
Above ground residue ratio	0.10	0.10	0.10	0.47	0.13	0.12	0.10
Below ground residue ratio	0.50	0.50	0.50	0.15	0.15	0.70	0.50
Residue N Concentration, kg N/kg ^4^							
Above ground	0.013	0.015	0.015	0.007	0.007	0.016	0.015
Below ground	0.007	0.015	0.015	0.010	0.010	0.010	0.015

DM = dry matter. ^1^ Mixture of timothy (70%) and red clover (30%). ^2^ Grass/legume silage information from Benchaar et al. [12]; other forages from Little et al. [17]. ^3^ Rotz and Muck [30]. ^4^ The mixed grass and alfalfa hays were wilted to approximately 90% dry matter prior to baling. ^5^ Little et al. [17].

**Table 4:** Greenhouse gas emission factors associated with crop production and processing, as well as imported feeds.

Item	Emission Factor	Unit	Reference
Crop Production			
Grass/legume silage (QC)	56.7	kg CO_2_e/ha	[31]
Corn silage and corn grain (QC)	161.0	kg CO_2_e/ha	[31]
Alfalfa hay (QC)	56.7	kg CO_2_e/ha	[31]
Barley grain and barley silage (AB)	124.6	kg CO_2_e/ha	[31]
Alfalfa hay (AB)	124.6	kg CO_2_e/ha	[31]
Mixed grass hay (AB)	124.6	kg CO_2_e/ha	[31]
Herbicide manufacture for corn silage and grain	0.696	kg CO_2_e/ha	[31]
Herbicide manufacture for barley silage and grain	1.334	kg CO_2_e/ha	[31]
N fertilizer manufacture	3.59	kg CO_2_e/kg N	[32]
P fertilizer manufacture	0.5699	kg CO_2_e/kg P_2_O_5_	[32]
Lime manufacture and transport	0.043	kg CO_2_e/kg CaCO_3_	[33]
Lime degradation	0.44	kg CO_2_e/kg CaCO_3_	[28]
Direct N_2_O emissions (QC)	0.013	kg N_2_O-N/kg N	[34]
Direct N_2_O emissions (AB)	0.0019	kg N_2_O-N/kg N	[34]
Leaching/run-off fraction	0.24	kg N/kg N	[28]
Indirect N_2_O emissions, leaching/run-off	0.011	kg N_2_O-N/kg N	[28]
Volatilization fraction	0.11	kg N/kg N	[28]
Indirect N_2_O emissions, volatilization (QC)	0.01	kg N_2_O-N/kg N	[28]
Indirect N_2_O emissions, volatilization (AB)	0.005	kg N_2_O-N/kg N	[28]
Purchased Feeds (QC Farm)			
Corn grain	1.29	kg CO_2_e/kg DM	[35]
Soybean meal and hulls (from QC)	0.56	kg CO_2_e/kg DM	[35]
Soybean meal and hulls (from ON)	0.58	kg CO_2_e/kg DM	[35]
Canola meal (from QC)	1.23	kg CO_2_e/kg DM	[35]
Canola meal (from SK)	0.29	kg CO_2_e/kg DM	[35]
Rumen inert fat	0.66	kg CO_2_e/kg DM	[36]
Sodium bicarbonate	0.44	kg CO_2_e/kg DM	[36]
Purchased Feeds (AB Farm)			
Corn grain	0.40	kg CO_2_e/kg DM	[35]
Soybean meal (from ON)	0.58	kg CO_2_e/kg DM	[35]
Canola meal (from AB)	0.46	kg CO_2_e/kg DM	[35]
Urea	3.30	kg CO_2_e/kg DM	[36]
Dicalcium phosphate	1.59	kg CO_2_e/kg DM	[36]
Magnesium oxide	1.05	kg CO_2_e/kg DM	[36]
Purchased Feeds (Both Farms)			
Beet pulp	0.56	kg CO_2_e/kg DM	[35]
Mineral/vitamin supplement	1.59	kg CO_2_e/kg DM	[36]
Calcium carbonate	0.013	kg CO_2_e/kg DM	[36]
Milk replacer	0.00134	kg CO_2_e/kg DM	[36]

AB = Alberta; DM = dry matter; QC = Quebec; SK = Saskatchewan.

**Table 5:** Diet ingredients for each animal category for the simulated Alberta dairy farm.

Animal Category	Calves	Heifers	Heifers	Bred Heifers	Lactating Cows ^1^		Dry Cows
Age Group/Scenario	0–3 Months	4–6 Months	7–16 Months	17–25 Months	SBM	CM	
	Feed Ingredients, % of DM						
Mixed grass hay	-	50.0	45.0	40.0	-	-	75.0
Barley silage	-	-	25.0	40.0	34.70	34.70	12.0
Alfalfa hay	-	25.0	-	-	10.00	10.00	-
Corn grain, ground	12.0	7.00	13.0	8.00	15.80	15.85	-
Barley grain, ground	12.0	6.50	9.00	5.00	15.80	15.85	7.00
SBM, 48% solvent extracted	2.25	5.00	3.50	3.00	10.20	0	2.50
CM, solvent extracted	2.25	5.00	3.50	3.00	-	13.00	2.50
Beet pulp, dehydrated	-	-	-	-	10.20	7.40	-
Urea	-	-	-	-	-	0.10	-
Mineral/vitamin supplement	1.50	1.50	1.00	1.00	1.36	1.36	1.00
Di-calcium phosphate	-	-	-	-	0.84	0.84	-
Calcium carbonate	-	-	-	-	0.76	0.76	-
Magnesium oxide	-	-	-	-	0.24	0.24	-
Milk replacer	70.0	-	-	-	-	-	-
	Diet Characteristics						
DMI, kg/day ^2^	2.33	4.70	8.30	12.54	25.22 (24.61)	24.38 (23.78)	12.42
NE_L_, Mcal/kg DM ^3^	0.55	1.34	1.38	1.32	1.51	1.55	1.07
TDN, % of DM ^4^	86.8	62.0	61.0	59.0	67.0	68.0	49.0
Crude protein, % of DM ^5^	18.7	16.4	13.3	13.2	20.0	18.5	11.1
Y_m_, % gross energy intake ^6^	0.00	6.30	6.30	6.30	5.85	5.85	6.30

CM = canola meal; DM = dry matter; SBM = soybean meal. ^1^ Lactation diets with SBM or CM. ^2^ Dry matter intake (DMI) for calves (0 to 3 months) and heifers (4 to 6 months) calculated based on intake requirements [27]; others estimated using Holos algorithms [28]; DMI of first lactation heifers in parenthesis. ^3^ Net energy of lactation values calculated based on feed ingredient composition tables [27]. ^4^ Total digestible nutrient (TDN) concentration for calves (0 to 3 months) and heifers (4 to 6 months) calculated based on feed ingredient composition tables [27]; others estimated using Holos algorithms [28]. ^5^ Crude protein for lactating cows from Oba et al. [19]; others calculated based on feed ingredient composition tables [27]. ^6^ Y_m_ based on IPCC [29].

**Table 6:** Greenhouse gas emission intensity of milk and meat produced by a dairy farm in Quebec using soybean meal (SBM) or canola meal (CM) sourced locally and nationally, with (CM_with_) and without (CM_without_) the enteric methane mitigating effect of CM, in the diets of the lactating cows.

Item	SBM Diet ^1^		CM_with_ Diet ^1^		CM_without_ Diet ^1^
	Local	National	Local	National	Local
Scenario number:	Q1	Q2	Q3	Q4	Q5
SBM source:	QC	ON	QC	QC	QC
CM source:	--	--	QC	SK	QC
Emission allocation, %					
Milk allocation	79.67	79.67	80.64	80.64	80.64
Meat allocation	20.33	20.33	19.36	19.36	19.36
Emission intensity					
kg CO_2_e/kg FPCM	0.909	0.919	0.978	0.853	1.017
kg CO_2_e/kg live weight	6.89	6.96	7.32	6.39	7.61
kg CO_2_e/kg carcass weight	11.48	11.61	12.20	10.64	12.69
kg CO_2_e/kg protein	30.10	30.43	31.81	27.74	33.08
kg CO_2_e/MJ energy	0.337	0.341	0.360	0.314	0.374

CM = canola meal; FPCM = fat and protein corrected milk standardized to 4% fat and 3.3% true protein; SBM = soybean meal. ^1^ CM and SBM were used in the diets of the calves and replacement heifers.

**Table 7:** Sources contributing to greenhouse gas emission intensity of fat and protein corrected milk (FPCM) for a dairy farm in Quebec using locally and national sourced soybean meal (SBM) or canola meal, with (CM_with_) and without (CM_without_) enteric methane mitigating effect, in the diets of the lactating cows.

Source	SBM Diet		CM_with_ Diet		CM_without_ Diet
	Local	National	Local	National	Local
*Scenario:*	Q1	Q2	Q3	Q4	Q5
g CO_2_e/kg FPCM					
CH_4_					
Enteric	367	367	336	336	375
Manure	54	54	55	55	55
N_2_O					
Manure, direct	35	35	35	35	35
Soils and cropping, direct	94	94	96	96	96
Soils and cropping, indirect	43	43	44	44	44
CO_2_					
Energy use	58	58	58	58	58
Imported feed	255	258	351	219	351
Feed transportation	4	11	4	10	4
Total	909	919	978	853	1017

**Table 8:** Greenhouse gas emission intensity of milk and meat produced by a dairy farm in Alberta using canola meal, with (CM_with_) and without (CM_without_) enteric methane mitigating effect, in the diets of the lactating cows.

Item	SBM Diet	CM_with_ Diet	CM_without_ Diet
Scenario:	A1	A2	A3
Emission allocation, %			
Milk allocation	82.34	82.86	82.86
Meat allocation	17.66	17.14	17.14
GHG intensity			
kg CO_2_e/kg FPCM ^a^	1.10	1.07	1.11
kg CO_2_e/kg live weight	8.07	7.79	8.12
kg CO_2_e/kg carcass weight	13.46	12.98	13.54
kg CO_2_e/kg protein	34.41	33.88	35.35
kg CO_2_e/MJ energy	0.399	0.386	0.402

^a^ Fat and protein corrected milk (FPCM) standardized to 4% fat, 3.3% true protein.

**Table 9:** Greenhouse gas emission intensity of fat and protein corrected milk (FPCM) produced by a dairy farm in Alberta using soybean meal (SBM) or canola meal, with (CM_with_) and without (CM_without_) enteric methane mitigating effect, in the diets of the lactating cows.

Source	SBM Diet	CM_with_ Diet	CM_without_ Diet
Scenario:	A1	A2	A3
g CO_2_e/kg FPCM			
CH_4_			
Enteric	442	404	450
Manure	74	74	74
N_2_O			
Manure, direct	45	49	49
Soils and cropping, direct	209	222	222
Soils and cropping, indirect	45	49	49
CO_2_			
Energy use	108	110	110
Imported feed	152	148	148
Feed transportation	24	12	12
Total	1101	1068	1115

## Data Availability

Data available by contacting K.A.B.
